# High-frequency, low-coverage “false positives” mutations may be true in GS Junior sequencing studies

**DOI:** 10.1038/s41598-017-13116-6

**Published:** 2017-10-23

**Authors:** Zhiliang Yang, Guilian Sun

**Affiliations:** Department of Pediatrics, the First Hospital of China Medical University, Shenyang, 110001 China

## Abstract

The GS Junior sequencer provides simplified procedures for library preparation and data processing. Errors in pyrosequencing generate some biases during library construction and emulsion PCR amplification. False-positive mutations are identified by related characteristics described in the manufacturer’s manual, and some detected mutations may have ‘borderline’ characteristics when they are detected in few reads or at low frequency. Among these mutations, however, some may be true positives. This study aimed to improve the accuracy of identifying true positives among mutations with borderline false-positive characteristics detected with GS Junior sequencing. Mutations with the borderline features were tested for validity with Sanger sequencing. We examined 10 mutations detected in coverages <20-fold at frequencies >30% (group A) and 16 mutations detected in coverages >20-fold at frequencies < 30% (group B). In group A, two mutations were not confirmed, and two mutations with 100% frequency were confirmed as heterozygous alleles. No mutation in group B was confirmed. The two groups had significantly different false-positive prevalences (p = 0.001). These results suggest that mutations detected at frequencies less than 30% can be confidently identified as false-positives but that mutations detected at frequencies over 30%, despite coverages less than 20-fold, should be verified with Sanger sequencing.

## Introduction

Next-generation sequencing (NGS) technologies are characterised by high-throughput sequencing data and are used widely in large-scale genome studies. Among the available NGS systems, Roche 454 platforms were the first to become commercially successful. Roche 454 genome sequencers (GS) include the GS 20 system, GS FLX Standard system, GS FLX Titanium system, and GS Junior System. These systems share the same core technology, which relies on emulsion PCR (emPCR)-based clonal amplification of a DNA library, attachment to micron-sized beads, and subsequent pyrosequencing. The GS Junior System can achieve comprehensive genome coverage with long 400-bp sequencing reads and quickly proceeds from DNA to base alignments with rapid sequencing runs and straightforward data analyses on the associated computer.

The 454 sequencers do produce false positives, however. Consequently, these systems require several reads to confirm whether the detected variations are true- or false-positive identifications. For example, in studies performed with the Reference Mapper software available in the GS Junior platform, each analysed allele should be observed in about 20–100 reads, and the variant frequency should not be less than 30%, according to the 454 sequencing system guidelines for amplicon experimental design (July 2011). In addition to a low number of reads and low frequency, other characteristics indicate whether a variant is a false positive, but the detection accuracy and sensitivity for a positive mutation depend primarily on the coverage and frequency. In a study using the 454 GS 20, the sensitivity for variant calls in shotgun library data was above 90% with a 20-fold range of coverage^1^.

Five studies that used the GS-FLX platform have provided a basis for the adopted cut-off values of coverage and frequency. The first study used GS Amplicon Variant Analyzer software and showed that the sensitivities differed for different exons even at the same simulated coverage. In addition, an average coverage range of 50-fold was sufficient to perform variant detection with a sensitivity of 99.8%^2^. The second study used a single nucleotide polymorphism discovery program called GIGABAYES. This group showed that a coverage range of 10- to 15-fold may be sufficient for resequencing applications but that higher coverage depths (50- to 60-fold) provide better alignment, assembly, and accuracy^3^. The third study used NextGENe^TM^ software, with parameters set to a sequence coverage range >30-fold and the percentage of heterozygous-allele calls to 40–60% to obtain reliable sequence data^4^. The fourth publication is a recent overview of factors that contribute to false-negative or false-positive variant calls with in-house–developed variant interpretation pipeline software. These authors suggested that when 99.99% detection sensitivity is required, cut-off values should be a 10-fold coverage range and 20% variation. In addition, the resulting variants with these values must be confirmed with Sanger sequencing, and a 5-fold coverage range is expected to be sufficient when screening for only homozygous variations^5^. The fifth study used Reference Mapper software, showing that this approach requires that reads harbouring the mutation should exceed 30% of the total reads^6^.

Six studies involving the GS Junior platform have provided a basis for the adopted standards of coverage and frequency. The first study used some web tools, including BWA-MEM, Cutadapt, VarScan, and a series of functions and filters that they programmed. These authors suggested thresholds for optimal accuracy and recommended a 38-fold coverage range to detect heterozygous alleles with a minimum 25% allele frequency for a sensitivity of 99.9%^7^. The second study used Amplicon variant analyser software. This group considered that a sample was truly mutated only when mutations were present in at least 1% of the consensual reads and at least 10 total reads were performed^8^. The third study did not mention the software used, and the investigators accepted the same standards used in the second study^9^. The final three of the six studies applied the Reference Mapper software. Of these, the fourth study showed that sequence variants were further prioritised according to the percentage (over 20%) of reads that contained a given variant^10^. The authors of the fifth study suggested a cut-off of 20-fold sequence coverage with 30% to 70% total variation in a heterozygous form and >90% in a homozygous form^11^. However, the authors of the sixth and final study indicated that only variants detected within a 10-fold coverage range with a 20% frequency should be considered when a detection power of 99.99% was required in the context of molecular diagnostics^12^.

In summary, the required coverage and frequency can differ according to the requirements of sensitivity, research purpose, and software used. That said, a 10-fold coverage range and 20% frequency may be the minimum requirements under special conditions.

Some mutations that are detected with only a few reads or at a low frequency and are considered false positives may be true positives. One study using the Reference Mapper software available in the GS Junior platform identified two variants with less than 20-fold depth or <30% total variation that were true-positive variants by Sanger sequencing verification^12^. In the last 3 years, mutation screening studies applying Reference Mapper software in the GS Junior platform also found several mutations with coverages less than 20-fold and frequencies over 30%, but no other false-positive characteristics, and Sanger sequencing also confirmed these to be true positives. In the present study, we collected 10 mutations with coverages <20-fold and frequencies >30% and 16 mutations with coverages >20-fold and frequencies <30%, and tested their validity with Sanger sequencing to determine the false-positive prevalence. We also investigated whether a false-positive detection was more likely to be identified by a coverage <20-fold or by a frequency <30% when the mutation had no other false-positive characteristics.

## Results

**Figure 1:**
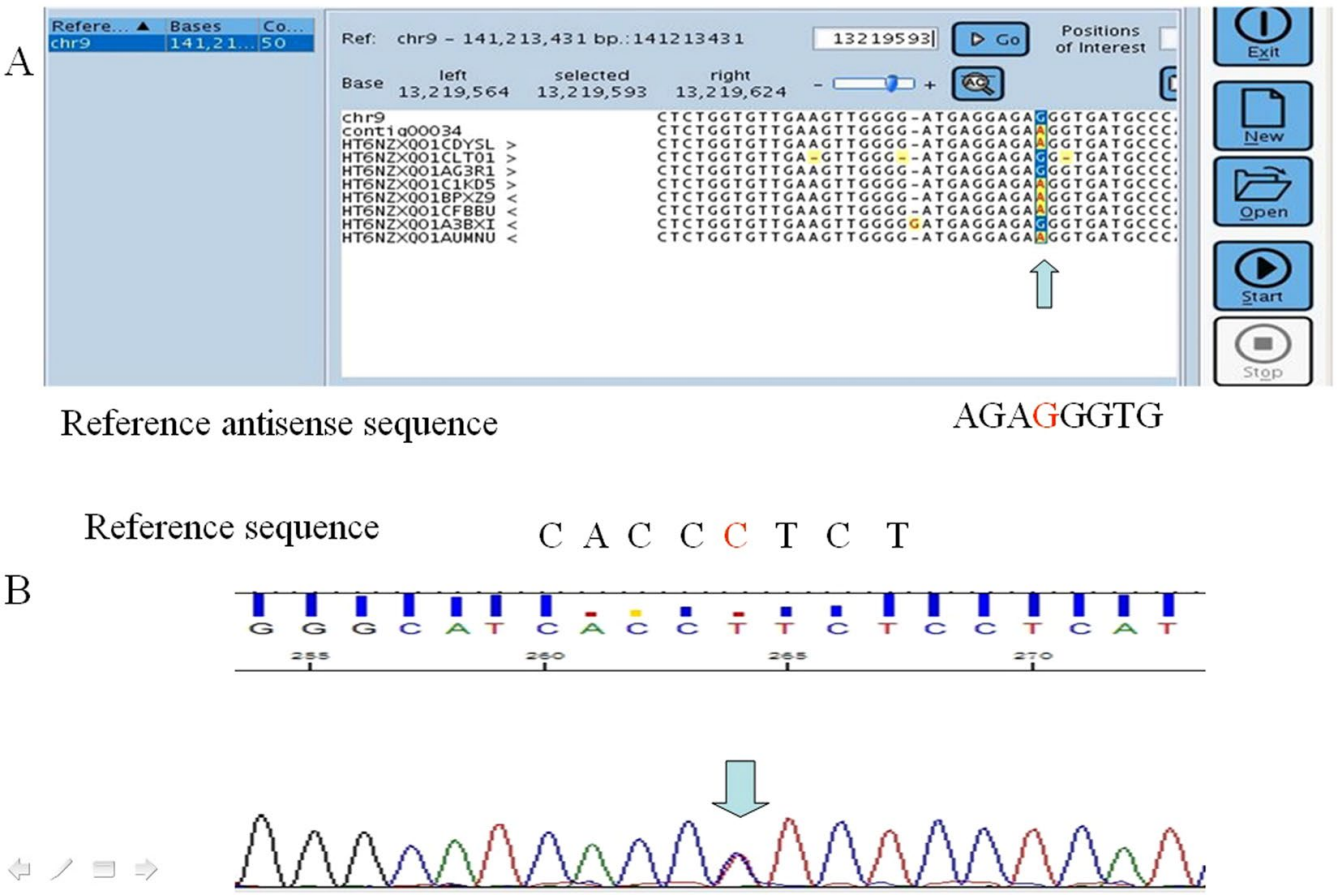
The electropherograms of representative A7. (**A**) The read image from the GS Junior system; the sequence is shown in sense sequence. There were three reads, and the reads showed a homologous mutation. A contig (from contiguous) is a set of overlapping DNA segments that together represent a consensus region of DNA. Contig 00053 was the GenBank accession number in the software for the reads. (**B**) The electropherogram from Sanger sequencing, and the sequence is shown in sense sequence. The mutation was verified to be heterozygous.

**Figure 2:**
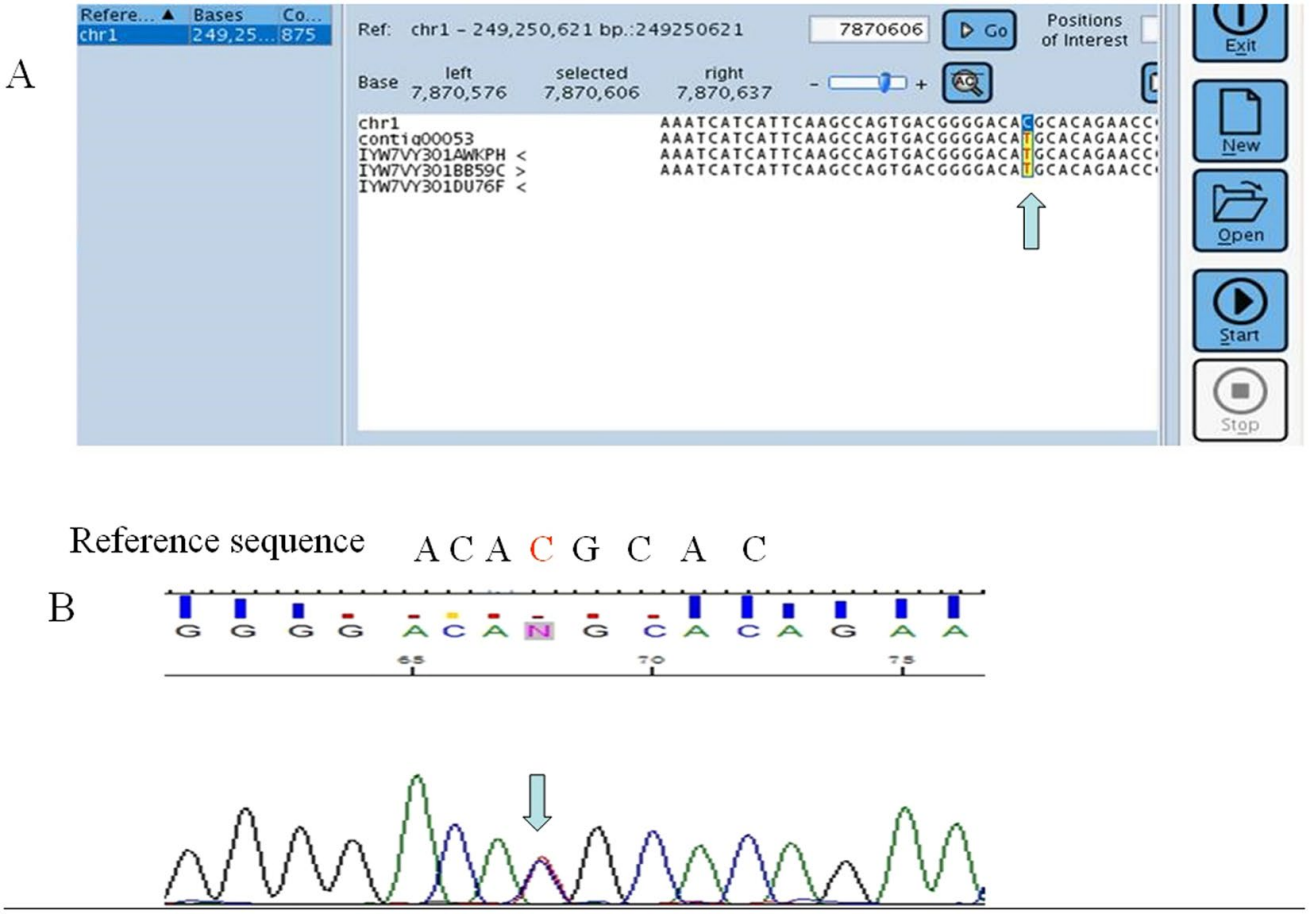
The electropherograms of representative A3. (**A**) The read image from the GS Junior system; the reads are shown in antisense sequences. There were six mutated reads among nine reads, and the mutations were considered false mutations according the characteristic criteria from the user manual. Contig 00034 was the GenBank accession number in the software for the reads. (**B**) The electropherogram from Sanger sequencing, and the sequence is shown in sense sequence. The mutation was verified as heterozygous.

In group A, mutations were detected in the 10 exons with a coverage range less than 20-fold and at frequencies greater than 30% (Table 1). None of the target regions were GC-rich sequences. Two mutations (A2 and A10) were not confirmed with Sanger sequencing, although one mutation appeared with 100% frequency. Two other mutations with 100% frequencies (A7 and A9) were confirmed to be heterozygous alleles (electropherograms of A7, Fig. 1), and they were also considered to be false positives in the study when calculating the prevalence of false-positive mutations. The electropherograms of representative A3 are shown in Fig. 2.

**Table 1:** Sanger sequencing verification results for mutations detected with GS Junior sequencing in group A, which were detected at low coverage depths but high frequency.

Codes	Gene and exon	Chr	Reference position	Length of PCR products	GC in PCR products (%)	Cdep	Vper (%)	Rnu	Vnu	RAA	VAA	Sanger sequencing result	Reported SNP
A1	MPDZ 42	9	13112024	475 bp	40.84	4	50	G	A	R	K	Confirmed	rs34605667
A2	MPDZ 39	9	13119582	637 bp	36.26	3	100	G	A	L	L	NOT Confirmed	N/A
A3	MPDZ 8	9	13219593	692 bp	33.82	9	66.7	C	T	L	F	Confirmed	rs3739757
A4	TIMELESS 13	12	56822105	456 bp	50.88	4	75	T	C	F	S	Confirmed	clinically significant
A5	NR1D1 2	17	38253630	523 bp	59.82	7	57	A	C	S	R	Confirmed	clinically significant
A6	SCTR 3	2	120236419	567 bp	59.26	5	60	G	A	P	L	Confirmed	clinically significant
A7	PER3 12	1	7870606	443 bp	48.68	3	100	C	T	R	C	Confirmed to be heterozygous	clinically significant
A8	PER2 11	2	239155102	452 bp	42.38	18	33.3	G	C	P	A	Confirmed	clinically significant
A9	SHANK2 22	11	70336398	472 bp	57.43	2	100	C	T	R	Q	Confirmed to be heterozygous	clinically significant
A10	TIMLESS 10	12	56824002	682 bp	53.11	12	50	G	A	A	T	NOT Confirmed	N/A

Chr: Chromosome; Cdep: Coverage depth; Vper: Variant percentage; Rnu: Reference nucleotide; Vnu: Variant nucleotide; RAA: Reference AA; VAA: Variant AA; N/A: Not applicable; SNP: single nucleotide polymorphism.

In group B, 16 mutations were detected with a coverage range greater than 20-fold and at frequencies less than 30% (Table 2). None of these 16 mutations were confirmed with Sanger sequencing.

**Table 2:** Sanger sequencing verification results for mutations detected with GS Junior sequencing in group B, which were detected at high coverage depths but low frequency.

Codes	Gene and exon	Chr	Reference position	Length of PCR products	GC in PCR products (%)	Cdep	Vper (%)	Rnu	Vnu	RAA	VAA	Sanger sequencing result
B1	MPDZ 12	9	13205082	343 bp	26.82	50	4	T	C	F	S	NOT Confirmed
B2	MPDZ 9	9	13217250	493 bp	28.74	32	13	—	A	D	V	NOT Confirmed
B3	MPDZ 11	9	13206034	487 bp	34.70	60	18	G	A	G	E	NOT Confirmed
B4	MPDZ 43	9	13110655	475 bp	40.84	80	8	T	C	S	P	NOT Confirmed
B5	MPDZ 23	9	13162715	353 bp	35.41	54	16	G	A	D	N	NOT Confirmed
B6	MPDZ 47	9	13106997	709 bp	38.22	42	11	A	G	T	T	NOT Confirmed
B7	MPDZ 29	9	13186367	552 bp	38.41	24	13	T	C	Y	H	NOT Confirmed
B8	MPDZ 17	9	13188931	451 bp	41.46	36	20	C	T	A	V	NOT Confirmed
B9	MPDZ 22	9	13168450	608 bp	33.22	54	19	A	G	I	V	NOT Confirmed
B10	MPDZ 36	9	13123284	455 bp	43.30	73	21	A	G	S	S	NOT Confirmed
B11	MPDZ 36	9	13123223	455 bp	43.30	48	10	A	G	T	A	NOT Confirmed
B12	PER2 19	2	239161869	369 bp	63.59	28	16	C	T	P	L	NOT Confirmed
B13	PER3 20	1	7897189	571 bp	43.05	34	6	A	G	T	A	NOT Confirmed
B14	MTNR1A 2	4	187455222	519 bp	52.27	68	15	C	T	R	H	NOT Confirmed
B15	PER2 11	2	239170928	452 bp	55.19	26	12	A	G	N	S	NOT Confirmed
B16	PER3 16	1	7886639	615 bp	48.48	24	8	T	C	V	A	NOT Confirmed

Chr: Chromosome; Cdep: Coverage depth; Vper: Variant percentage; Rnu: Reference nucleotide; Vnu: Variant nucleotide; RAA: Reference AA; VAA: Variant AA.

The prevalences of false-positive mutations were 40% in group A and 100% in group B. The false-positive prevalences were significantly different between the two groups (p = 0.001).

## Discussion

The 454 GS Junior sequencers may produce false positives, which could lead to misleading conclusions. To avoid such pitfalls, only mutations that fulfil the standards for a positive identification are used for result analyses. Thus, the minimum read depth and minimum percentage of mutated reads are set to eliminate random sequencing errors. In some studies, only the variants detected in over 10-fold or 30-fold coverage ranges^3,4,8,9^ or variants with mutations detected in over 30% of the total reads^4,6^ have been used to draw a conclusion. However, in a study that used the GS Junior System and GS Reference Mapper software, some true variants were skipped because they were detected in coverages less than 20-fold or at frequencies less than 30%^12^. In the present study, eight true variants were considered false positives based on the cut-off values; however, these were later confirmed as true positives with Sanger sequencing. In contrast, none of the mutations detected at frequencies less than 30% were confirmed with Sanger sequencing. These results confirmed the importance of being aware of different potential generation mechanisms of artifacts, which can result in false-positive calls.

Bias can have an influence at several steps, including amplicon-based library preparation, fragment enrichment, and sequencing. False-positive mutations may be generated by chimeric sequences that form during the PCR amplification step. Chimeras arise through a PCR-mediated recombination, which creates an artificial, false-positive haplotype. Chimera formation may be induced by high cycle numbers, high initial template concentrations, and polymerases^13–16^. Reported chimera formation rates range from 1% to 5% across a variety of polymerases^14–16^, and rates are much higher in some other studies^17–19^. PCR biases can also be induced by suboptimal primers^20^. Consequently, to reduce biases, optimum template concentrations, fewer cycles, high-fidelity polymerases, and optimal primers require consideration.

Templates with high GC base compositions are frequently difficult to amplify with PCR. GC contents over 65% may induce aberrant amplification from non-target regions, producing multiple bands on gel electrophoresis^21^. Unusual sequence characteristics, like high GC content or the presence of repetitive elements, can also cause poor enrichment in sequence capture enrichment approaches, reducing sequencing efficiency^4^. A high GC content in the 5′-untranslated region of an exon can also hinder efficient target sequence capture^22^. On the other hand, the 454 NGS sequencing has some technical limits in detecting mutations in homopolymers^23^. In the present study, GC content was calculated in the PCR products of the amplicon libraries and in the exons of the custom-designed SeqCap EZ Choice libraries. We found no GC effects in the regions targeted in this study.

**Figure 3:**
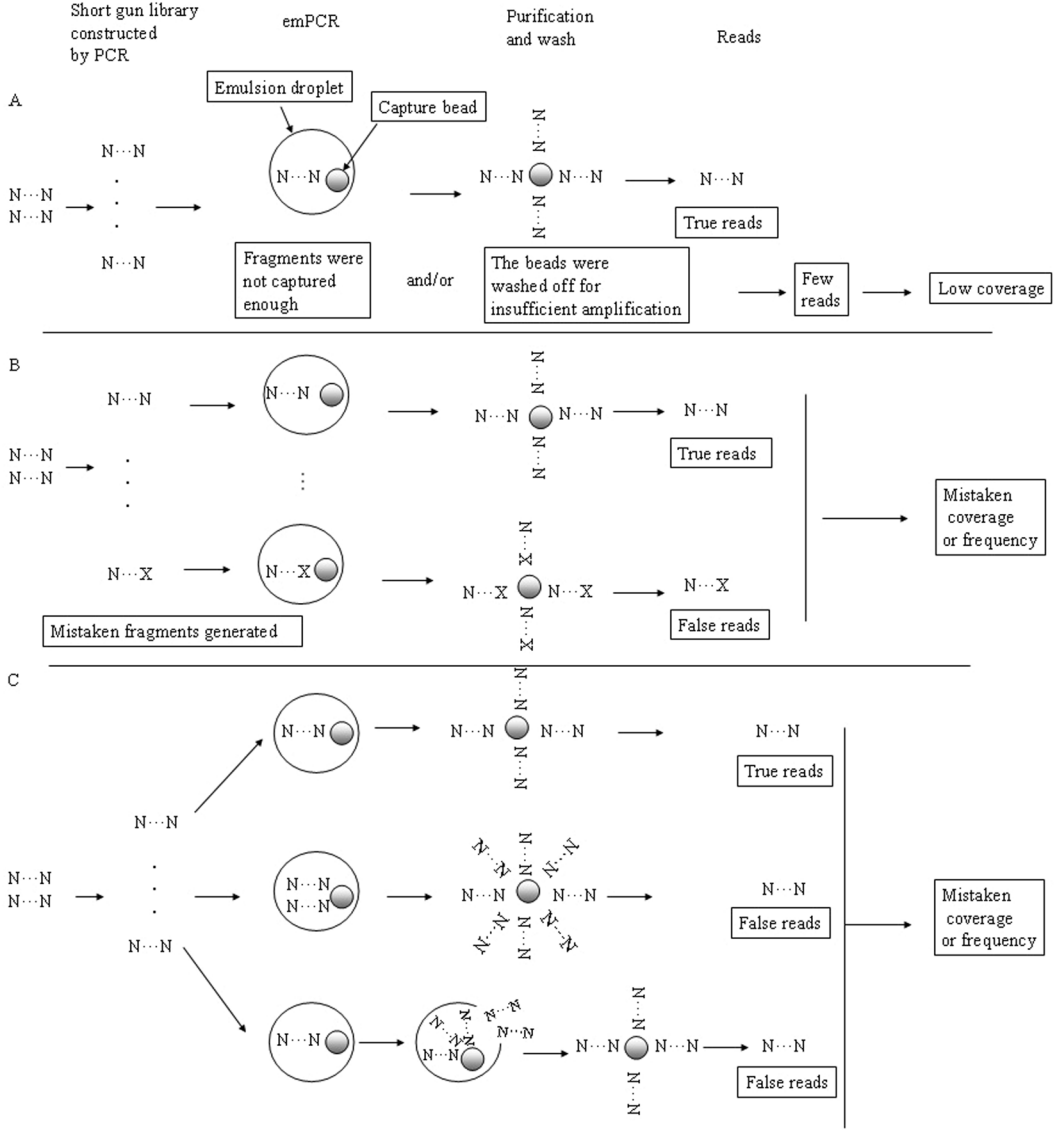
Possible mechanisms of false-read generation during library construction and emulsion PCR. One bead should be one unique read. (**A**) Clonal amplification of single DNA on one bead in one droplet, which would result in a unique read. If capture beads did not capture enough fragments or the beads were washed off for insufficient amplification, the result may be few final reads. (**B**) Some mistaken fragments generated during library construction may be captured and amplified, whereas the true fragments of the same position may fail to be amplified, which could result in some kind of high-frequency false positive. If both artificial and natural duplicates were captured and amplified, the result can be some type of low-frequency false positives. (**C**) If several fragments were captured by one bead in one droplet or some droplets were broken during the process of emPCR, the result also may be a false positive with low frequency.

Enrichment approaches that capture single DNA fragments in solution have the advantage that many regions can be targeted in parallel; however, some targeted regions may not be captured and other, unwanted regions may be. When the beads fail to sufficiently capture one type of fragment or are washed off the fragment, that sequence will be insufficiently amplified and few reads achieved (Fig. 3A). Alternatively, when mistaken fragments generated during the PCR amplification are captured and amplified but the true fragments at the same position are not sufficiently amplified, the result may be detection of a high-frequency, false-positive mutation (Fig. 3B). Only 5-fold coverage has been identified as sufficient for screening homozygous variations^5^. In another study, though, a mutation with a cut-off of 20-fold sequence coverage and >90% frequency was considered homozygous^11^, and the remaining <10% frequency reads were considered false reads. However, when only the mutated fragments are amplified and the normal fragments are not, the final results may appear to indicate a homozygous variant. In the present study, three types of 100% frequency variants were detected with GS Junior sequencing; one was not confirmed and two were confirmed as heterozygous alleles with Sanger sequencing (Table 1).

Biased amplifications in the emPCR process might result in producing artificial duplicate reads. Artificial duplicates can be generated when several fragments are captured by one bead in one droplet or when some droplets are broken during the emPCR process^24^. The presence of artificial duplicates thus may result in the detection of a low-frequency false-positive mutation (Fig. 3C). In this study, the 16 mutations in group B were low-frequency false positives that could not be confirmed with Sanger sequencing. Similarly, a previous study that used the GS amplicon variant analyser software and the 454 FLX platform reported two mutations that appeared at frequencies of 18.6% and 22.9%, which could not be confirmed with Sanger sequencing^25^. Two other studies using Reference Mapper software and GS Junior sequencing reported two heterozygote mutations detected at frequencies of 22.6% with a 53-fold coverage and 21.3% with a 61-fold coverage, and neither could be confirmed with Sanger sequencing^10^. In addition, variants detected at frequencies of about 30% could not be confirmed with Sanger sequencing^11^. Thus, the characteristic of ‘detection at low frequency’ appears to be a strong indicator for a false-positive mutation.

The GS 454 sequencing platform was reportedly not good at detecting indel mutations in a study using the NGS platform to detect such mutations^26^. In our studies, we did not detect any indels, but we cannot attribute this outcome to the platform because our samples may have harboured no indels. The sequencing errors were not only the result of random chance but also of systematic errors in the technique itself or artifacts, especially in homopolymeric regions. Despite the occurrence of false positives in studies using the Reference Mapper software with the GS Junior platform, the GS Junior platform remains a powerful method for large-scale genome studies. Even when the detected mutations must be verified with Sanger sequencing, the GS Junior platform requires less time than Sanger sequencing to perform the same work.

One shortcoming of this study is the small number of enrolled mutations, and more mutations with coverage <20 and frequency >30% to ensure data validation are needed. In addition, not all types of DNA regions were covered, such as GC-rich regions. We found that even when the quality control met standards recommended in the manufacturer’s manual, some mutations occurred when the coverage was <20 and the frequency >30%. The results of this study indicated that mutations detected over a few reads (less than 20-fold coverage) and at high frequencies (over 30%) should be verified with Sanger sequencing in studies that use Reference Mapper software and GS Junior sequencing. Because of unavoidable differences in using apparatuses and variable proficiency in manipulating them, researchers will benefit from establishing their own thresholds for true mutations.

## Materials and Methods

### DNA templates

The present research was performed in accordance with the ethical guidelines of the 1975 Declaration of Helsinki and permitted by the Ethics Committee of Jichi Medical University and the Ethics Committee of the First Hospital of China Medical University. Participants or their relatives provided written informed consent to participate in the study.

For this study, we identified 26 target regions in 8 genes (groups A and B) that were shown in two earlier studies to harbour mutations. One study was a mutation screening study in exons of circadian-relevant genes using a custom-designed SeqCap EZ Choice library^27^, and the other was a mutation screening study in exons of *MPDZ* (multiple PDZ domain protein) using rapid amplicon library (shotgun). All participants fulfilled the diagnostic criteria for Autism Spectrum Disorder as listed in the Diagnostic and Statistical Manual of Mental Disorders: Fourth Edition (DSM-IV). All 137 patient samples were sequenced for mutation screening of MPDZ, and 28 of these samples were sequenced for mutation screening of circadian-relevant genes. All patients had intellectual disability (ID), and their IQs ranged from 14 to 75.

The statistical significance for detected mutations was analysed between the patient and control groups. The hypothesised effects of the detected mutations on respective protein functions were analysed using the Polymorphism Phenotyping v2 (PolyPhen-2) prediction tool (http://genetics.bwh.harvard.edu/pph2/), SIFT (http://sift.jcvi.org/), and Mutation Taster (mutation t@sting, http://www.mutationtaster.org/). If there was no statistical significance between the groups for a detected mutation, then that mutation was considered a polymorphism.

All gene sequences were obtained from http://www.ncbi.nlm.nih.gov/nucleotide/. Lymphocyte samples were obtained from patients who provided informed consent. Samples were transfected with the Epstein–Barr virus (to establish lymphoblasts) and cultured *in vitro*. Genomic DNA was extracted from lymphoblasts with the salting-out method. DNA concentrations were determined with a NanoDrop 2000 spectrophotometer (Thermo Fisher Scientific).

### Amplicon rapid library (shotgun) preparation

Primers were designed to amplify exons and their vicinities for all 46 exons of the *MPDZ* gene to yield amplicons of 250–700 bp. All PCR was carried out under the following conditions: 25 ng of human genomic DNA, 0.1 μM of each primer, 1.5 μl of 10 × PCR buffer (Takara, Shiga, Japan), 1.2 μl of 200 mM dNTPs (Takara, Shiga, Japan), and 0.1 μl of rTaq DNA polymerase (Takara, Shiga, Japan). The final volume was adjusted to 15 μl with water. Thermocycling conditions were one initial incubation at 94 °C for 3 min, followed by 36 cycles at 94 °C for 30 s, the appropriate annealing temperature for 30 s or 45 s, and 72 °C for 30 s. A final extension step at 72 °C for 10 min was added at the end of the last cycle. The reactions were performed in a Gene Amp PCR system 9700 (PE Applied Biosystems). The products were then purified and ligated with different multiplex identifiers (MIDs; we used12 MIDs; Titanium Rapid Library MID adaptor; Roche, Pleasanton, CA, USA). The MIDs enabled the use of the pooled sequences as a library, according to the GS Junior Titanium Series Rapid library (shotgun) Preparation Manual, June 2012.

### NimbleGen SeqCap EZ Choice library preparation

Exons of targeted genes were identified in the reference human genome, version hg19 (http://www.ensembl.org/). We constructed the SeqCap EZ library according to the NimbleGen Sequence Capture Custom Design “Guide to Submitting Your Target Sequence”. Briefly, 500 ng of genomic DNA was nebulised for 1 min with 30 psi of pressure. The nebulised DNA was purified with Agencourt AMPure XP beads (Beckman Coulter, Fullerton, CA, USA). Different MIDs (12 MIDs; Titanium Rapid Library MID adaptor; Roche, Pleasanton, CA, USA) were ligated to the fragmented DNAs to construct a library, according to the NimbleGen SeqCap EZ Library LR User’s Guide (Version 2.0, November 2011).

### Emulsion PCR and GS Junior Sequencing

The libraries were pooled in equimolar amounts and combined with capture beads at a ratio of two molecules from each DNA library per capture bead. The pooled DNA was then amplified with emulsion PCR (emPCR). The bead-attached DNAs were denatured, eluted, and quantified with the provided bead counter. All were performed according to the GS Junior Titanium Series emPCR (Lib-L) Manual (June 2012).

A total of 500,000 enriched DNA beads were mixed with Packing Beads. Then, the Pico Titer Plate was sequentially loaded with Prelayer Beads, DNA-Packing Beads, Postlayer Beads, and PPiase Beads. Finally, the Pico Titer Plate was mounted in the 454 GS Junior Sequencer, and the program was run in full processing mode for shotgun sequencing, according to the GS Junior Titanium Series Sequencing Method Manual (June 2012). The resulting reads were aligned, and variants were compared to the reference genome with the 454 integrated software (GS Reference Mapper; Roche, Pleasanton, CA, USA).

### Sanger sequencing

Primers (Table 3) were designed to target exons that harboured mutations identified in previous studies. PCR was performed to amplify each exon and its neighbouring introns. The PCR products were purified with the MinElute® PCR purification kit (Qiagen) and checked with 2% agarose gel electrophoresis. Sequencing was performed by applying the same forward and reverse primers that were used for PCR amplification. We used the BigDye® terminator sequencing kit, version 3.1 (Life Technologies), and a 3730XL DNA analyser (Life Technologies). The sequencing results were interpreted with Sequence Scanner, version 1.0 (Applied Biosystems).

**Table 3:** The primers used in Sanger sequencing verification.

Codes	Gene and exon	Forward primer (5′ to 3′)	Reverse primer (5′ to 3′)
A1	MPDZ 42	CTTCGTGGCTTTGAACACAG	CTAGGGCTTCTAGGGTTGATAG
A2	MPDZ 39	TACACGCTTCTACTCAGTG	TTTTGCCATGTTGCCAAGGC
A3	MPDZ 8	TATGTCCTGATTGGTAACTC	ATGACCCACCTTACAGAGAG
A4	TIMELESS 13	ACATATCTCCAGATGAGGCTG	TGTACAGCTTAAGTTCATCT
A5	NR1D1 2	GATGAGCACAGTGGCACCCTA	CTATTCTGGCTTCAGGGATT
A6	SCTR 3	TAGACAGGATAGGTTAATGAT	CTTCAGCTAGGAGCTCTCTT
A7	PER3 12	GTGATTACTGTGCTTCAGTC	GCAATAGTAGTAATAATAATG
A8,B15	PER2 11	GCAGTGGCTTTGAAGAAGTG	CTTCCTGCCACCAATCTGGA
A9	SHANK2 22	TGTGATGTGTAATTTGCCAT	GATTATTTCCCATTAAGTGA
A10	TIMLESS 10	CATCATTACACTCCAGCCTG	GTGATGATGCAGCTGTTGGT
B1	MPDZ 12	CAGATTCTCATGTAGGAGTTC	CCATAAGACCACTTAGTCAC
B2	MPDZ 9	GAATTAAAGTAGGCCTGTGGC	CCATCTTCGATTCTCCAACT
B3	MPDZ 11	GGTCTTACTGTCAATTCCCTG	CACTTCTTTACTCGCCTTGG
B4	MPDZ 43	ATTTCAGTGGCTGGTAGCAG	TCATGTACTGCTAAGGCCTG
B5	MPDZ 23	GAAGTTTGGATTCTCTGCTG	CTTTGCTCTAGTCCTCCCTGA
B6	MPDZ 46	GAGCAGTGTGTGTGTACAAG	GGTAGATTGCCAAATTGAGGC
B7	MPDZ 29	CAGGAAACCAGTCCTGATG	GGAACATGGACTCTACTTGG
B8	MPDZ 17	GGCACTTGAGACAATTACTC	CGAATGGAGCTAGTAATCACG
B9	MPDZ 22	CATAGTACTCTCCCTGAGAG	CGTTTGCATCTCAAACAGAAG
B10,B11	MPDZ 36	CCTCAGAGTCAATCTGTGTG	CACCAGTGTAGCAGAATATTGC
B12	PER2 19	CCTGTCATGGCATTCATGC	GACTGCAAACCTGGCACTTCT
B13	PER3 20	GAAGTGCTATTCCTAGATGACG	GGTCGTGTTCTTGCATGATC
B14	MTNR1A 2	GATCTACTCGTGCACCTTCG	CTCTGAACTTCATTGGCCTG
B16	PER3 16	TCTGTGCTAGGCTAATGAAG	CTGATGGAGAGATGCTGAAAC

### Data analysis

Statistical significance was analysed with Fisher’s exact test. Analyses were performed with IBM SPSS statistics 21 software, and a p-value less than 0.05 was used to define statistical significance.

### Data availability

The datasets generated during and/or analysed during the current study are available from the corresponding author on reasonable request.
